# Factors Predetermining Increased Aqueous Humour Flare in Long-Term Glaucoma Treatment

**DOI:** 10.1155/2020/7345687

**Published:** 2020-03-23

**Authors:** G. Pakuliene, L. Kuzmiene, I. Januleviciene

**Affiliations:** Department of Ophthalmology, Lithuanian University of Health Sciences, Kaunas, Lithuania

## Abstract

Glaucoma patients often require long-term or even lifelong medical antiglaucomatous treatment. Benzalkonium chloride (BAK) is the most frequently used preservative in medical glaucoma treatment. Laser flare photometry is the noninvasive quantitative measurement of anterior chamber protein level and helps tracking intraocular inflammation. The purpose of our study was to evaluate the ocular aqueous humour flare in glaucoma patients, scheduled for cataract surgery without any other ocular diseases, and the association with pseudoexfoliation (PEX) syndrome, number of medications used, and BAK. A prospective case-control age- and gender-matched study, including open-angle glaucoma patients (>2 years of treatment) with cataract, matched with cataract patients with no other ocular pathology (control group). We found that the aqueous humour flare was higher in the glaucoma group than in the control group. PEX syndrome increased the aqueous humour flare independently from glaucoma diagnosis. The number of used antiglaucomatous medications correlated moderately with the aqueous humour flare. The BAK index showed weak positive correlation with aqueous humour flare. A variety of factors can affect aqueous humour flare increase, including PEX syndrome, medical substance used to treat glaucoma, number of different medications, and presence of BAK. The combination of these factors is of key importance to long-term glaucoma treatment.

## 1. Introduction

Glaucoma patients often require long-term or even lifelong medical antiglaucomatous treatment [1]. Daily administration of ocular drops interferes with ocular surface integrity and increases the risk for adverse effects [2]. Both medical substance and preservative can contribute to toxicity-related ocular adverse effects [2]. This is even more important, if the patient requires ocular surgical treatment, after the history of long-term glaucoma medical treatment [3].

Benzalkonium chloride (BAK) is the most frequently used preservative in medical glaucoma treatment [4]. The inflammatory properties of BAK are very well presented by the contribution to dry eye disease and a variety of inflammatory cytokines found on ocular surface [5, 6]. Experimental animal studies show that topical administration of BAK on the ocular surface increases the corneal permeability and can lead to BAK presence intraocularly [7, 8]. BAK acts as a detergent and emulsifier, proposing the risk of intraocular inflammation, hence found intraocularly [6, 9, 10].

Laser flare photometry is the noninvasive quantitative measurement of anterior chamber protein level [11]. The technology allows tracking intracameral protein increase and inflammation [11]. The subclinical increase in aqueous humour flare using laser flare photometry in pseudoexfoliation syndrome (PEX) patients was observed back in 1992 [12]. Later on, the developing technology allowed to identify subtle differences in aqueous humour flare increase between different glaucoma patients, different medications, or with preservative presence in medications [13–16]. However, these studies confined to only one mentioned causative factor.

The purpose of our study was to evaluate ocular aqueous humour flare in glaucoma patients, scheduled for cataract surgery without any other ocular diseases, and the association with pseudoexfoliation (PEX) syndrome, number of medications used, and BAK.

## 2. Materials and Methods

We conducted a prospective case-control age- and gender-matched study. The case-control ratio was 1 : 2. The Kaunas Regional Biomedical Ethics Committee approved all study procedures. All of the participants signed an informed consent form. The study adhered to the tenants of Declaration of Helsinki.

The open-angle glaucoma group (treated for >2 years) with cataract was matched to the control group of cataract patients with no other ocular pathology. Inclusion criteria: >18 years old, intraocular pressure (IOP) <21.0 mmHg, no ocular hyperaemia or medication intolerance, and no previous ocular surgery.

The methods included full ophthalmic evaluation, Goldmann applanation tonometry for IOP, and ocular aqueous humour laser flare and cell photometry (Kowa FM-700 ver. 2.01.200000, Japan). Aqueous humour flare was analysed without pupil dilation [17, 18]. Ten measurements were obtained from each eye, and marginal values were eliminated to increase accuracy. Flare count was presented as photon count per millisecond (pc/ms). We additionally analysed the groups divided by presence of PEX syndrome, number of glaucoma medications used daily, and BAK index. The BAK index was calculated by adding up the used antiglaucomatous medications' BAK concentrations once or twice, depending on the daily prescription.

We used the following formula:

(1) $$\begin{array}{c} \mathrm{Index}\;\left(\text{BAK}\right)=\;Xx1\;+\;Yx2\;+\;Zx2\;+\;Qx0. \end{array}$$

where *X*, *Y*, *Z*, and *Q* are BAK concentrations in medications, and it is multiplied by prescription once (1) or twice (2) daily, (0) if not prescribed.

To detect the difference of 3 pc/ms between the groups, we needed at least 20 participants in each group (*α* = 0.05, *β* = 0.1, power 90%).

All of the participants answered the Ocular Surface Disease Questionnaire (OSDI©, Allergan, Ireland) for ocular surface complaints. We also performed Schirmer's test and tear break-up time (TBUT) for objective ocular surface evaluation. Schirmer's test was performed by adding a Schirmer's paper strip in the inferior fornix. Five minutes later, the strip was inspected for the length of moisture (mm) in the paper strip. This test demonstrated basal and reflex tear secretion. TBUT was performed by adding fluorescein dye in the inferior fornix of the eye. The ocular surface was observed under slit lamp with cobalt blue light. TBUT was measured in seconds until the tear film broke.

Statistical analysis was performed with SPSS v23.0 program package. We used Student's *t* test for two normally distributed independent samples and Mann–Whitney *U* test for two nonparametric independent samples. Spearman's rank correlation coefficient was used for nonparametric ranking correlations. We considered *p* > 0.05 statistically significant.

## 3. Results and Discussion

The glaucoma group included 22 subjects and 44 subjects in the control group. Demographic data are presented in Table 1.

### 3.1. Aqueous Humour Flare

**Figure 1:**
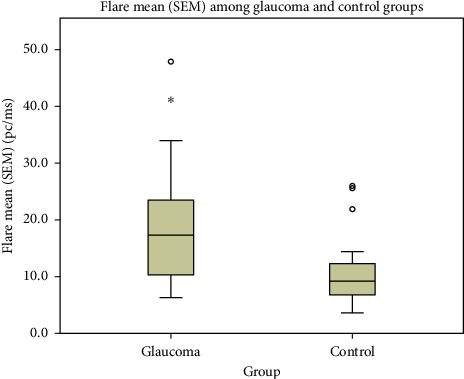
The graph shows aqueous humour flare mean (SEM) among glaucoma and control groups. The glaucoma group showed significantly higher aqueous humour flare than that of the control group (*p* < 0.001, Mann–Whitney *U* test). PEX syndrome and aqueous humour flare.

The aqueous humour flare mean (SEM) in the glaucoma group was 18.9 (2.2) pc/ms and median 17.3 pc/ms, and accordingly 10.0 (0.76) pc/ms and median was 9.2 pc/ms in the control group (*p* < 0.001, Mann–Whitney *U* test) (Figure 1). There was no significant correlation between IOP and aqueous humour flare (*p* > 0.05, Spearman's rho).

**Figure 2:**
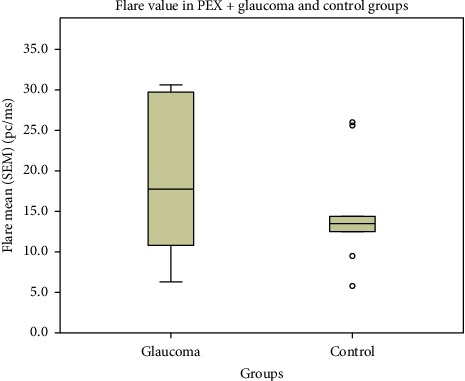
Flare value among (PEX−) control and glaucoma groups. The mean values did not differ significantly; however, the glaucoma (PEX−) group showed a higher aqueous humour flare tendency than the control group.

**Figure 3:**
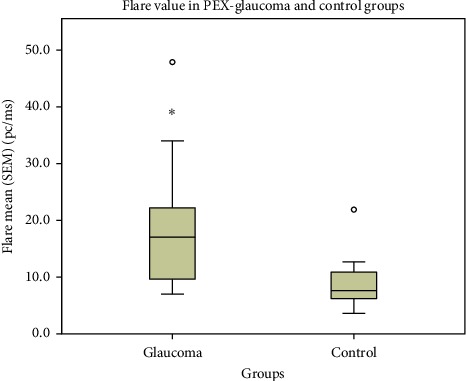
Flare value among PEX-control and glaucoma groups. The glaucoma group showed significantly higher aqueous humour flare mean values than the control group. Ocular surface's subjective and objective evaluation.

PEX was found in 10 glaucoma and 9 control subjects. Aqueous humour flare mean (SEM) in the glaucoma (PEX+) group (*n* = 10) was 18.7 (2.8) pc/ms and median 17.8 pc/ms, while in the control group (PEX+) (*n* = 9) it was 14.8 (2.3) pc/ms and median 13.5 pc/ms, (*p*=0.234, Mann–Whitney *U* test) (Figure 2). Aqueous humour flare mean (SEM) in the glaucoma group (PEX−) (*n* = 12) was 19.0 (3.4) pc/ms and median 17.0 pc/ms, and accordingly 8.6 (0.7) pc/ms and median 7.6 pc/ms in control (PEX−) (*n* = 35) (*p* < 0.001, Mann–Whitney *U* test) (Figure 3).

OSDI© scores were similar among control and glaucoma groups. The mean (SEM) total OSDI© score was 19.17 (2.9) in the control group and 22.19 (2.9) in the glaucoma group (*p*=0.174, Mann–Whitney *U* test). Schirmer's test value mean (SEM) was 12.95 (1.3) in the control group and 10.05 (2.0) in the glaucoma group (*p*=0.222, Student's *t* test). TBUT value mean (SEM) was 8.70 (0.8) and median 7.0 in the control group and mean (SEM) 8.50 (1.1) and median 7.0 in the glaucoma group (*p*=0.784, Mann–Whitney *U* test).

### 3.2. BAK Index and Aqueous Humour Flare

**Figure 4:**
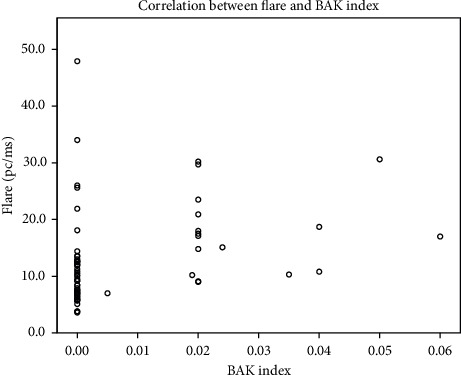
Correlation between flare and BAK index in the glaucoma group; a weak positive correlation (Spearman's rho = 0.390, *p*=0.001).

We found weak positive correlation between aqueous humour flare and BAK index (Spearman's rho = 0.390, *p*=0.001) (Figure 4); number of medications and aqueous humour flare.

**Figure 5:**
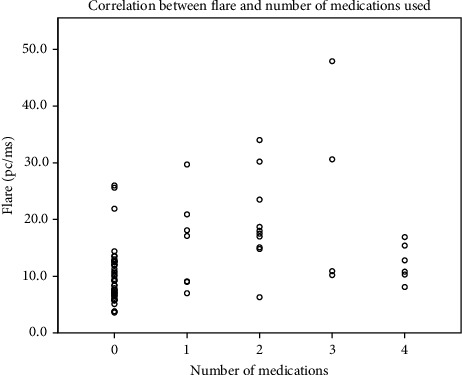
Correlation between flare and the number of different medications used in the glaucoma group; a moderate positive correlation (Spearman's rho = 0.495, *p* < 0.001).

We found moderate positive correlation between aqueous humour flare and the number of different types of antiglaucomatous medications used (Spearman's rho = 0.495, *p* < 0.001) (Figure 5). The majority of our participants with glaucoma (*n* = 19) received prostaglandin treatment with either latanoprost, bimatoprost, travoprost, or tafluprost; beta-blockers (timolol) (*n* = 13), alpha agonists (brimonidine) (*n* = 4), and carbonic anhydrase inhibitors (dorzolamide or brinzolamide) (*n* = 8). Monotherapy was prescribed to 7 of participants with glaucoma, 5 of which received only the prostaglandin inhibitor, and the remaining two received timolol.

Several studies showed increased aqueous humour flare in patients with PEX syndrome independently from glaucoma diagnosis [12, 15, 19]. Older of these studies did not show the aqueous humour flare difference between non-PEX controls and non-PEX glaucoma patients; Kahloun et al. were able to identify the difference, and our study results were consistent with their findings [12, 15, 19]. Kahloun et al. excluded participants who were treated with prostaglandins due to the ability of altering blood-aqueous barrier [13–15, 20]. Arcieri et al. investigated the aqueous humour flare 4 weeks after prostaglandin analogues prescription but did not find significant aqueous humour flare increase [14]. We did not exclude participants with prostaglandins; however, our results did not differ much from Kahloun et al.'s findings [15]. The majority of our overall participants with glaucoma received treatment with the prostaglandin analogues. Most of the participants, who received antiglaucomatous monotherapy, received the prostaglandin analogue. We also found moderate positive correlation between the number of different antiglaucomatous medications and aqueous humour flare value. This would mean that, if prostaglandins were important in aqueous humour flare findings, the influence was not isolated.

We found that aqueous humour flare and BAK index had a weak positive correlation. Stevens et al. in their one-month long study observed that prescribing timolol *de novo* increased the aqueous humour flare; the BAK-preserved timolol increased the aqueous humous flare more than BAK-free timolol [16]. We also found a moderate positive correlation between aqueous humour flare and number of different medications prescribed. Our study presented long-term combined antiglaucomatous medications' relation with aqueous humour flare. It is obvious that there is no single causative factor for aqueous humour flare increase in long-term medical treatment perspective. Modification of medical substances is a difficult task; however, modifying the preservative is much more possible.

One of the advantages in our study was that we excluded patients with ocular hyperaemia and medication intolerance, which prevented significant inaccuracy in our findings. The OSDI© questionnaire, TBUT, and Schirmer's test results were similar among both groups, which allowed decreasing misinterpretation of our results due to dry eye disease. Aqueous humour flare photometry required clear media and no ocular surface inflammation for accurate flare measurement, and any ocular surface alterations could lead to false results [11].

The other advantage of our study was that participants with glaucoma had already received antiglaucomatous treatment for more than two years and were tolerating it well. This means that the participants, who received prostaglandin analogues, had already been past the transient ocular hyperaemia window [21, 22]. In contrast, Cellini et al. prescribed *de novo* treatment with prostaglandin analogues to their participants and found that after three months, the aqueous humour flare increased; however 6 months later, the flare values slightly decreased, except for the bimatoprost group [13].

As for shortcomings, due to relatively small study sample, we could not identify each antiglaucomatous medication's effect on aqueous humour flare separately, only the combined effect.

## 4. Conclusions

Numerous factors can affect aqueous humour flare increase, including PEX syndrome, medical substance used to treat glaucoma, number of different medications, and presence of BAK. The combination of these factors is of key importance to long-term glaucoma treatment. Further long-term studies are needed to evaluate the effect of flare increase and other causative factors impact on glaucoma treatment.

## Figures and Tables

**Table 1:** Demographic data of participants in control and glaucoma groups.

Demographic data	Glaucoma	Control	*p*
Number of participants	22	44	—
Male/female ratio (%)	32/68	32/68	—
Age mean (SEM) (years)	72.6 (8.2)	74.7 (8.9)	>0.05 (Student's *t* test)
IOP mean (SEM) (mmHg)	16.4 (0.6)	15.0 (0.4)	>0.05 (Mann–Whitney *U* test)

## Data Availability

The authors confirm that the data supporting the findings of this study are available within the article and supplementary materials.

## Supplementary Materials

Supplementary Materials Provided Supplementary material is a table of all antiglaucomatous medications, used by patients in our study. Alongside, we provided brand names of medications and BAK concentrations in each medication, as provided in information leaflets. BAK concentrations were used to calculate the BAK index.

## References

[1] Kalouda P., Keskini C., Anastasopoulos E., Topouzis F. (2017). Achievements and limits of current medical therapy of glaucoma. *Glaucoma Surgery*.

[2] Liang H., Brignole-Baudouin F., Pauly A., Riancho L., Baudouin C. (2011). Polyquad-preserved travoprost/timolol, benzalkonium chloride (BAK)-preserved travoprost/timolol, and latanoprost/timolol in fixed combinations: a rabbit ocular surface study. *Advances in Therapy*.

[3] Boimer C., Birt C. M. (2013). Preservative exposure and surgical outcomes in glaucoma patients. *Journal of Glaucoma*.

[4] Steven D. W., Alaghband P., Lim K. S. (2018). Preservatives in glaucoma medication. *British Journal of Ophthalmology*.

[5] Malvitte L., Montange T., Vejux A. (2007). Measurement of inflammatory cytokines by multicytokine assay in tears of patients with glaucoma topically treated with chronic drugs. *British Journal of Ophthalmology*.

[6] Aguayo Bonniard A., Yeung J. Y., Chan C. C., Birt C. M. (2016). Ocular surface toxicity from glaucoma topical medications and associated preservatives such as benzalkonium chloride (BAK). *Expert Opinion on Drug Metabolism & Toxicology*.

[7] Brignole-Baudouin F., Desbenoit N., Hamm G. (2012). A new safety concern for glaucoma treatment demonstrated by mass spectrometry imaging of benzalkonium chloride distribution in the eye, an experimental study in rabbits. *PLoS One*.

[8] Droy-Lefaix M. T., Bueno L., Caron P., Belot E., Roche O. (2013). Ocular inflammation and corneal permeability alteration by benzalkonium chloride in rats: a protective effect of a myosin light chain kinase inhibitor. *Investigative Opthalmology & Visual Science*.

[9] Brycki B., Małecka I., Koziróg A. (2017). Synthesis, structure and antimicrobial properties of novel benzalkonium chloride analogues with pyridine rings. *Molecules*.

[10] Lockington D., Macdonald E. C. A., Stewart P., Young D., Caslake M., Ramaesh K. (2012). Free radicals and the pH of topical glaucoma medications: a lifetime of ocular chemical injury?. *Eye*.

[11] Tugal-Tutkun I., Herbort C. P. (2010). Laser flare photometry: a noninvasive, objective, and quantitative method to measure intraocular inflammation. *International Ophthalmology*.

[12] Küchle M., Nguyen N. X., Horn F., Naumann G. O. H. (1992). Quantitative assessment of aqueous flare and aqueous “cells” in pseudoexfoliation syndrome. *Acta Ophthalmologica*.

[13] Cellini M., Caramazza R., Bonsanto D., Bernabini B., Campos E. C. (2004). Prostaglandin analogs and blood-aqueous barrier integrity: a flare cell meter study. *Ophthalmologica*.

[14] Arcieri E. S., Pierre Filho P. T. P., Wakamatsu T. H., Costa V. P. (2008). The effects of prostaglandin analogues on the blood aqueous barrier and corneal thickness of phakic patients with primary open-angle glaucoma and ocular hypertension. *Eye*.

[15] Kahloun R., Attia S., Ksiaa I. (2016). Anterior chamber aqueous flare, pseudoexfoliation syndrome, and glaucoma. *International Ophthalmology*.

[16] Stevens A. M., Kestelyn P. A., De Bacquer D., Kestelyn P. G. (2012). Benzalkonium chloride induces anterior chamber inflammation in previously untreated patients with ocular hypertension as measured by flare meter: a randomized clinical trial. *Acta Ophthalmologica*.

[17] Karaca I., Güven Yılmaz S., Palamar M., Ateş H. (2019). Effect of tropicamide on laser flare meter measurements in patients with pseudoexfoliation. *Ocular Immunology and Inflammation*.

[18] El-Harazi S. M., Ruiz R. S., Feldman R. M., Chuang A. Z., Villanueva G. (2002). Quantitative assessment of aqueous flare: the effect of age and pupillary dilation. *Ophthalmic Surgery Lasers*.

[19] Küchle M., Nguyen N. X., Hannappel E. (1994). Tyndallometry with the laser flare cell meter and biochemical protein determination in the aqueous humor of eyes with pseudoexfoliation syndrome. *Ophthalmologe*.

[20] Selen F., Tekeli O., Yanık Ö. (2017). Assessment of the anterior chamber flare and macular thickness in patients treated with topical antiglaucomatous drugs. *Journal of Ocular Pharmacology and Therapeutics*.

[21] Chen J., Dinh T., Woodward D. F. (2005). Bimatoprost: mechanism of ocular surface hyperemia associated with topical therapy. *Cardiovasc Drug Reviews*.

[22] Yanagi M., Kiuchi Y., Yuasa Y. (2016). Association between glaucoma eye drops and hyperemia. *Japanese Journal of Ophthalmology*.

