# Supplementary material for: Factors Predetermining Increased Aqueous Humour Flare in Long-Term Glaucoma Treatment
Source: J Ophthalmol. 2020 Mar 23;2020:7345687. doi: 10.1155/2020/7345687 (PMC7125493; doi:10.1155/2020/7345687)
Supplement: Supplementary Materials — Provided Supplementary material is a table of all antiglaucomatous medications, used by patients in our study. Alongside, we provided brand names of medications and BAK concentrations in each medication, as provided in information leaflets. BAK concentrations were used to calculate the BAK index. [file 7345687.f1.docx]

**Supplementary Materials**

| Medication | Brand name | Preservative | BAK  Concentration % |
| --- | --- | --- | --- |
| Latanoprost | Xalatan | BAK | 0.02 |
| Travoprost | Travatan | sofZia® | 0.01 |
| Bimatoprost | Bimican | BAK | 0.005 |
| Tafluprost | Taflotan | - | 0 |
| Timolol | Arutimol 0.5 | BAK | 0.01 |
| Brimonidine | Luxfen | Purite® | 0 |
| Brinzolamide | Brinzolamide Elvim | BAK | 0.01 |
|  | Optilamid | BAK | 0.015 |
|  | Azopt | BAK | 0.01 |
| Dorzolamide | Nodofree | - | 0 |
|  | Trusopt | BAK | 0.0075 |
| Combined: |  |  |  |
| Latanoprost/Timolol | Xalacom | BAK | 0.02 |
| Travoprost/Timolol | Duotrav | Polyquad® | 0 |
| Bimatoprost/Timolol | Ganfort | BAK | 0.05 |
| Dorzolamide/Timolol | Cosopt | BAK | 0.0075 |
| Brinzolamide/Timolol | Azarga | BAK | 0.1 |
| Brinzolamide/Timolol | Simbrinza | BAK | 0.03 |

Table 2. Concentrations of BAK, as provided in package leaflet of each medication. Concentrations were used to calculate BAK index.
